# Rapid Northward Expansion of the Blacklegged Tick, *Ixodes scapularis* , in Response to Climate Change

**DOI:** 10.1111/gcb.70591

**Published:** 2025-11-06

**Authors:** Jacob R. Westcott, Joseph J. Bowden, Jade Savage, Karen M. Doody

**Affiliations:** ^1^ School of Science and the Environment Grenfell Campus, Memorial University of Newfoundland Corner Brook Newfoundland Canada; ^2^ Natural Resources Canada Canadian Forest Service Corner Brook Newfoundland Canada; ^3^ Department of Biological Sciences Bishop's University Sherbrooke Quebec Canada

**Keywords:** climate adaptation, deer tick, ecological niche models, Ixodidae, macroecology, species distribution model, tick‐borne pathogens, vector‐borne disease

## Abstract

Climate change rapidly drives species range dynamics, prompting many terrestrial organisms to shift northward to higher latitudes and forcing new species–species and species–environment interactions. The blacklegged tick, *Ixodes scapularis* , a biological vector of human pathogens including *Borrelia burgdorferi* (the bacteria causing Lyme disease), is undergoing rapid and persistent expansion into Canada, exposing new human populations to zoonotic diseases. Here, we used an ensembled forecasting approach to construct niche models of *I. scapularis'* current and future distribution and to identify the environmental drivers of habitat range. Georeferenced occurrence points were acquired from community science programs (eTick and iNaturalist) between 2017 and 2022 in Canada and the United States. We also collected high‐resolution environmental data using a spacing of approximately 1 km. We carried out 4704 model iterations across two datasets, 12 algorithms, and 10 climate profiles using 40 environmental variables. We extrapolated select models over three time periods, 2011–2040, 2041–2070, and 2071–2100, across two projected climate scenarios, SSP5‐8.5 and SSP3‐7.0, incorporating 2094 future outcomes of *I. scapularis* distribution. Our ensembles (AUC: 0.9565 ± 0.0065; TSS: 0.8435 ± 0.0155; Kappa: 0.819 ± 0.014) identified temperature, precipitation, biomass production (NPP), length of the growing season, climate moisture index, and number of yearly degree days as the variables that best explained *I. scapularis* distribution. Further changes to these climate conditions will result in continued *I. scapularis* range expansion, with, at the highest estimate, an increased niche area of ~248% (447,532 km^2^ to 1,556,760 km^2^) and, at the lowest estimate, by ~205% (409,475 km^2^ to 1,247,689 km^2^) before the turn of the century. These distributional niche changes coincide with a northern latitude limit reaching as far as ~48° N by 2040, ~50° N by 2070, and ~52° N by 2100. These findings highlight the invasive potential of *I. scapularis* , with implications for public health and changing ecosystem dynamics.

## Graphical abstract

The blacklegged tick, *Ixodes scapularis* , can transmit human diseases such as Lyme disease and is exposing new human populations to this risk due to its rapid expansion into Canada. We used community science data (eTick and iNaturalist) to understand the environmental factors responsible for the distribution of *I. scapularis* and used these to predict where we will likely see populations of ticks in the future. Our modeling predicts that further climate changes could result in continued northward range expansion and significantly increased suitable area by the turn of the century, highlighting the invasive potential of *I. scapularis* , with implications for public health and ecosystems.

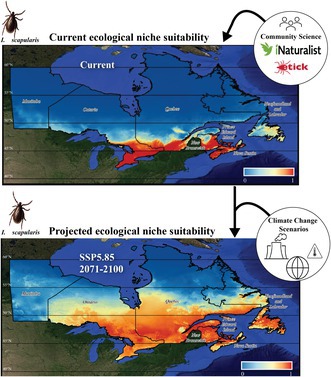

## 1 Introduction

Climate change is having severe and widespread impacts on people, biodiversity, and ecosystems (IPCC 2023). Over the last 50 years, global surface temperatures have risen faster than any other half‐century period over the last 2000 years (IPCC 2023). As such, climate change is redistributing species across the landscape (Chen et al. 2011), with over half the species assessed by the Intergovernmental Panel on Climate Change (IPCC) moving poleward (IPCC 2023). This phenomenon results in increased novel species interactions, with implications for ecology, economics, and human health (Alexander et al. 2015; Gilman et al. 2010; Wikel 2018).

Climate has a particularly strong effect on terrestrial arthropods (insects, spiders, ticks etc.). The external environment dictates the physiology of terrestrial arthropods (Harrison et al. 2012; MacDonald et al. 2020) thereby driving where and how these organisms can live. Climate change is influencing their body sizes, distributions, phenologies, genotypes, abundances, development rates, and even host preferences (e.g., Backus et al. 2021; Levi et al. 2015; Nuttall 2021; Ogden et al. 2021; Porretta et al. 2013). For arthropods that serve as major vectors of zoonotic diseases (e.g., ticks, mosquitoes), climate change carries not only implications for their own biological and ecological systems but also for the economy as well as human, animal, and environmental health.

The blacklegged tick, *Ixodes scapularis* , is of growing concern in Canada as a vector of several human pathogens (Eisen and Eisen 2018). In North America, *I. scapularis* is the primary vector of the spirochete bacterium *Borrelia burgdorferi* sensu stricto, the etiological agent responsible for Lyme borreliosis, commonly known as Lyme disease (Belli et al. 2017). Lyme disease is North America's most common vector‐borne infectious disease (Ginsberg et al. 2021; Levi et al. 2015; Rosenberg et al. 2018). In recent years, other emergent pathogens vectored by *I. scapularis* (e.g., *Anaplasma phagocytophilum* —the bacteria causing anaplasmosis, *Babesia microti*—the parasite causing babesiosis) have been on the rise in eastern North America (Public Health Ontario 2024).

In North America, *I. scapularis* has historically been endemic to the eastern United States. However, climate change is pushing the geographical limits of *I. scapularis* northward into Canada. The leading edge of *I. scapularis'* range is expanding at approximately 48 (±2) km per year (Clow et al. 2017; Talbot et al. 2019), nearly three times faster than the average animal species (IPCC 2023). Tick range expansion can be assisted over shorter distances (up to tens of kms) by animals, such as deer and smaller mammals, with birds as the leading cause of long‐range expansion (hundreds–thousands of kms; Halsey et al. 2018; Keirans et al. 1996, Ogden, Lindsay, et al. 2008). Indeed, several passerine migrant birds, lagomorphs (e.g., snowshoe hares) and other small mammals in central and eastern Canada serve as competent reservoirs for *B. burgdorferi* (Scott et al. 2018). Scott et al. (2018) also showed that *Haemaphysalis leporispalustris* (which can be positive for *B. burgdorferi* ) and *I. scapularis* parasitize many of these species during northward migration into Canada. More recently, Zinck and Lloyd (2022) showed that several medium and small mammals and bird species are likely competent reservoirs for *B. burgdorferi*. For instance, Scott et al. (2018) tested 18 tick species widely distributed across Canada, finding 83% were positive for *B. burgdorferi* , with 13 species capable of biting humans.

Long‐term habitat suitability and establishment of tick populations are more dependent on specific environmental conditions rather than movement into novel ranges. If environmental conditions support the overwintering and permanent establishment of *I. scapularis* (Burrows et al. 2021) and the immigration rates of infected larvae and nymphs reach a sufficient threshold, there is typically a five‐year lag between tick establishment and *B. burgdorferi* invasion (Ogden et al. 2013).

*I. scapularis* is sensitive to environmental conditions such as temperature, humidity, precipitation, land cover type, and canopy cover or understory density (Leighton et al. 2012; Thomas et al. 2020; Slatculescu et al. 2020). Even during winter, which is warming rapidly in the north, increasing temperatures mean increasing activity of this tick (Ferguson et al. 2024) with the potential to increase the host‐seeking activity period. The complex interplay between ticks and their environment has necessitated the development and application of sophisticated modeling techniques to identify the geographic distribution of ticks and to forecast potential habitat suitability (Slatculescu et al. 2020). Ecological niche models (ENM) effectively develop predictions of current and future species distributions (Zhu et al. 2021). Consensus (ensemble) modeling approaches are frequently employed to integrate outcomes from individual ENM modeling algorithms, thereby generating more robust and explicit results by incorporating multiple modeling parameters (Zhu et al. 2021; Melo‐Merino et al. 2020).

Macroecological models require broad spatial and often temporal scale data that can be provided by open community science platforms such as iNaturalist and eTick. These platforms have benefited greatly from carefully vetted community science contributions, which represent a cost‐effective means for improving and validating modeling approaches (e.g., Wang et al. 2018; Saunders et al. 2020; Hallman and Robinson 2024). Here, we develop ensembled ENMs using the most comprehensive dataset of blacklegged tick records in eastern North America collected via community science to identify the environmental factors influencing the current distribution of *Ixodes scapularis* and employ these models to predict how future climate scenarios will drive changes in the distribution of this species in eastern Canada over the next century.

## 2 Materials and Methods

### 2.1 Tick Occurrence and Environmental Data

We focused our study on eastern Canada (including the province of Manitoba) and assembled extensive community‐sourced tick occurrence data and bioclimatic variables that cover Manitoba, Ontario, Quebec, Nova Scotia, New Brunswick, Prince Edward Island (PEI), and Newfoundland and Labrador (NL). To capture the full environmental gradient linked to the distribution of *I. scapularis* , we also gathered occurrence and environmental data from thirty‐seven states in the eastern United States that contained empirical records of *Ixodes scapularis* at the time of the analysis (Table S1). A total of 12,859 *I. scapularis* georeferenced and verified occurrence records were compiled from two community science image‐based data sources: iNaturalist and eTick, between 2017 and 2022 (eTick 2022; iNaturalist 2022). While the provinces joined the eTick program in different years, they had a minimum of 3 years of data, with the exception of PEI, which had 2 years (eTick 2022). Most data were collected during spring and early summer (April, May, and June) and in the fall (October and November). The two platform datasets include all life stages of *I. scapularis* ; however, adult specimens comprised the majority of recorded observations. We curated the datasets by first merging and removing duplicate records. Additionally, we removed records where data entries exhibited positional inaccuracies > ~1 km or displayed other apparent errors or inconsistencies. We also excluded records from the iNaturalist dataset with coordinate systems classified as private or obscured. After corrections, 11,504 records remained within the dataset. To mitigate potential over‐sampling bias from the datasets that can cause model coefficients to disproportionately become more representative of environmental conditions from regions of higher sampling (Inman et al. 2021), we tested several occurrence distribution patterns and their impact on model performance to address class imbalances and spatial bias. We used a spatial thinning process, frequently implemented to improve model performance (Aiello‐Lammens et al. 2015), where we removed points within 50 m of impervious surfaces and retained only one record per 1 km gridded cell across the study area. As a result, the number of *I. scapularis* was reduced from 11,504 to 3500 (2155 from Canada). Tick pseudo‐absence data followed Barbet‐Massin et al. (2012), who provide the basis for optimal quantity and distribution of pseudo‐absence points.

Environmental data (42 different predictors; Table S2) was collected from two sources: *Chelsa‐Bioclim*+ (Brun et al. 2022; Karger et al. 2023; Karger, Lange, et al. 2021; Karger, Wilson, et al. 2021) and *AdaptWest* (AdaptWest Project 2022; Mahony et al. 2022; Wang et al. 2022, 2016). The Chelsa‐Bioclim+ dataset was used as the foundational dataset for this study, and we used the AdaptWest dataset to cross‐validate the results. The Chelsa‐Bioclim+ dataset is based on the GFDL‐ESM4 model and includes historical data from 1981 to 2010. The AdaptWest rasters were generated using *ClimateNA v7.3* with historic climate normals from 1991 to 2020. Both datasets use a spatial resolution of ~1 km.

### 2.2 Occurrence Models

We used an ensemble (consensus) approach to model species distribution. This approach is based on a succession of individual models and is founded on the theory that individual predictors are replicas of potential states of the real distribution (Marmion et al. 2009).

The data (georeferenced records and environmental variables) was analyzed using R (R Core Team 2023) in RStudio (RStudio Team, 2020) using the following packages: *sdm* (Naimi and Araujo 2016), *rgdal* (Bivand et al. 2022), *raster* (Hijmans 2022), *plyr* (Wickham 2011), *usdm* (Naimi et al. 2014), *caret* (Kuhn 2022), *biomod2* (Thuiller et al. 2022), *ENMeval* (Kass et al. 2021), *gridExtra* (Auguie and Antonov 2017), *dplyr* (Wickham et al. 2022), *CENFA* (Rinnan and Lawler 2019), and *corrplot* (Wei and Simko 2021).

To produce the ensemble of models we used 12 algorithms: Generalized Linear Models (GLM), Generalised Additive Models (GAM), Generalised Linear Models with Polynomial Regression (GLMPoly), Multivariate Adaptive Regression Splines (MARS), Multiple Discriminant Analysis (MDA), Flexible Discrimination Analysis (FDA), Recursive Partitioning (RPART), Classification and Regression Trees (CART), Boosted Regression Trees; otherwise known as Generalised Boosted Regression Models (BRT/GBM), Random Forests (RF), Support Vector Machines (SVM), Radial Basis Function Network (RBF), and Multilayer Perceptron (MLP). Applicable algorithms had hyperparameters selected and tuned using the *caret* package (Kuhn 2022; Yang and Shami 2020); a 5‐fold cross‐validation procedure was run eight times with a tuning length of 50; the best parameters were chosen based on the highest receiver operating characteristic curve (ROC) value.

Algorithms were trained and produced models through the *sdm* package (Naimi and Araujo 2016). We used an 80/20 data partition, training‐test split of the data using subsampling, bootstrapping, and 5‐fold cross‐validation (Naimi and Araujo 2016) which resulted in multiple models per algorithm. We produced twenty‐eight models per algorithm, partitioned using three resampling techniques: 4 subsampled models, 4 bootstrapped models, and 20 cross‐validated models.

Each time the script was processed the ensuing models used different environmental variables to further capture the broad spectrum of environmental factors (e.g., temperature, humidity, habitat vegetation characteristics, moisture; Berger et al. 2014; Ginsberg et al. 2020; Lubelczyk et al. 2004) that mediate *I. scapularis* distribution. Environmental variables were tested for multicollinearity in each individual model, with pairwise exclusion of highly correlated variables using variance inflation factor (VIF) and selected in a stepwise manner, using Pearson's correlation and based on a correlation threshold of < 0.8 and a VIF threshold of < 10 between each paired variable (Dormann et al. 2012; Naimi et al. 2014; Naimi 2023). We also reviewed the literature to identify and retain environmental variables that could be responsible for driving tick distribution.

When we assessed the model performances using the dependent test data, we considered three metrics: Area Under the ROC Curve (AUC) and True Skill Statistic (TSS) for predicting the probability of species presence/absence or habitat suitability, and Cohen's kappa (Kappa) to evaluate uncertainty or prevalence (Steen et al. 2021). Models with metrics below threshold values of AUC < 0.80 (Li et al. 2023), TSS < 0.70 (e.g., Thuiller et al. 2019), and Kappa < 0.6 (e.g., as suggested in McHugh 2012) were removed. The top‐performing model from each algorithm was retained; in cases where multiple top models demonstrated comparable performance metrics, they were averaged. We then combined the models to produce five ensembles. Because each ensemble represents a habitat suitability prediction, we subsequently averaged the five ensembles with each other to produce a single suitability map.

### 2.3 Forecasting Tick Distributions

The models from each algorithm used in constructing the historical ensemble were also extrapolated and ensembled to project the future distribution of *I. scapularis* under two climate scenarios (SSP3‐7.0 and SSP5‐8.5) across three time periods (2011–2040, 2041–2070, and 2071–2100). SSP5‐8.5 outlines a high‐emissions trajectory driven by heavy reliance on fossil fuels and an energy and resource‐intensive global economy with rapid socioeconomic growth (Meinshausen et al. 2020). In contrast, SSP3‐7.0 represents a medium‐high emissions pathway characterized by slow economic and population growth, high levels of material consumption, unequal resource distribution, and low prioritization of environmental issues, making adaptation and mitigation efforts challenging (Riahi et al. 2017; Meinshausen et al. 2020). Thirty‐year averages were used across the three time periods to reduce noise and variability in the climate projections. We selected these scenarios understanding that they represent the medium‐high, and the high reference scenarios, where there is limited to no additional climate policy, respectively. This precautionary approach was taken to mitigate potential risks associated with model uncertainty in these predictive analyses. This process was first achieved using the Chelsa‐Bioclim+ dataset and subsequently emulated with the AdaptWest dataset. We used a chi‐square test of homogeneity to test for significant change in the proportions of suitable habitat (land area) over time and employed a Bonferroni correction for multiple (post hoc comparison) tests.

Ensembles are comprised of grid cells with each cell representing approximately 1 km^2^ of ground area. Each cell is assigned a predictive value between zero and one, which indicates the niche suitability for *I. scapularis*. The following classifications were used to indicate the probability of niche suitability: unsuitable niche (0–0.1), low suitability niche (0.1–0.3), medium suitability niche (0.3–0.6), high suitability niche (0.6–0.8), and optimal suitability niche (0.8–1).

## 3 Results

To identify the environmental variables pertinent to driving *I. scapularis* distribution, we conducted 4704 model iterations across two datasets, employing up to 12 algorithms. We investigated 10 climate profiles and considered a total of 42 environmental variables. Under the two projected climate scenarios (SSP5‐8.5 and SSP3‐7.0), we projected a total of 2094 future predictions of *I. scapularis* distribution. After evaluating all the models, the Chelsa‐Bioclim+ ensembles consisted of 183 models employing 11 algorithms. The final ensembled results demonstrated an average performance with AUC: 0.963, TSS: 0.859, and Kappa: 0.833. Figure S1 illustrates the performance comparisons of the algorithms among the retained models.

**FIGURE 1:**
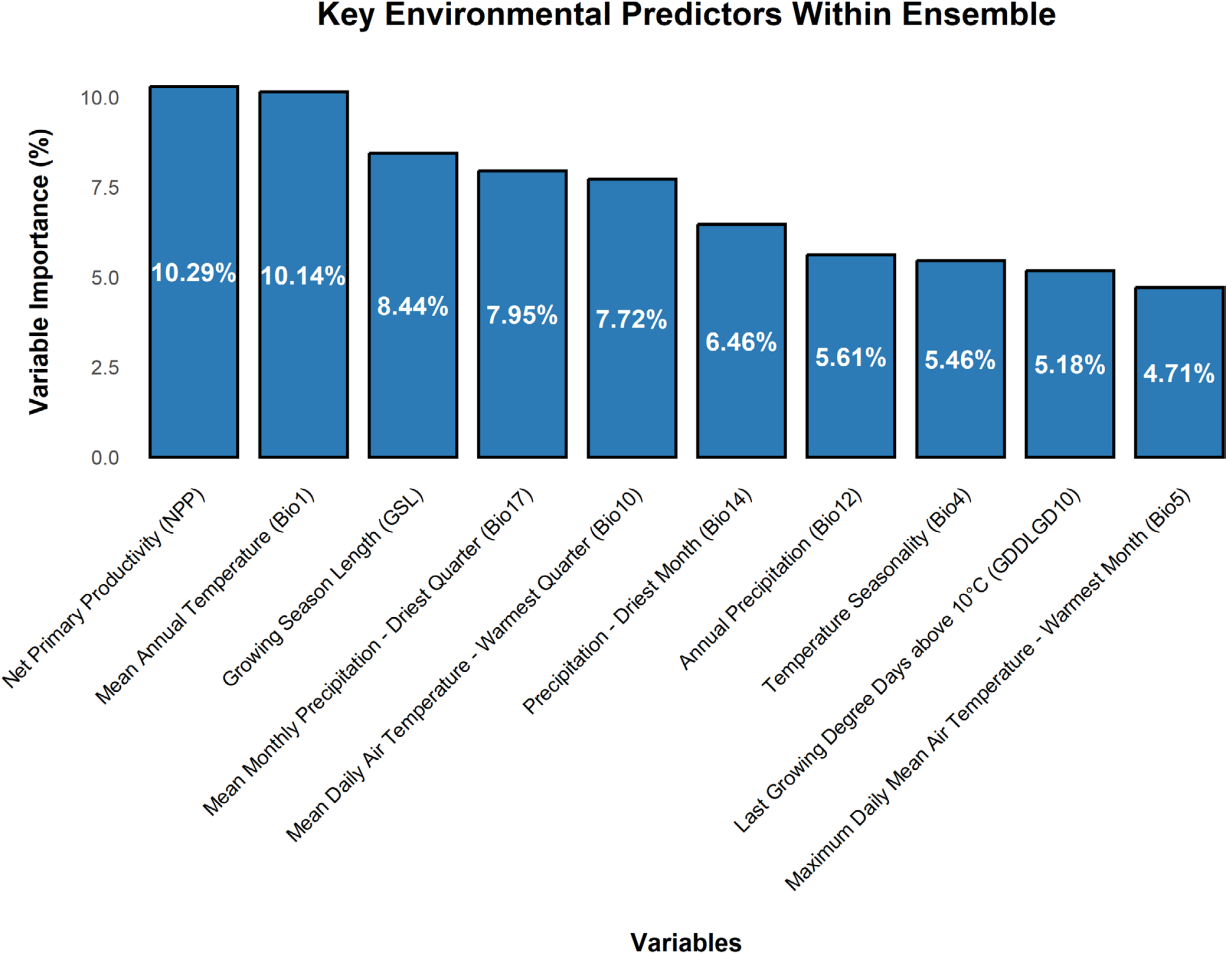
The most influential Chelsa‐Bioclim+ environmental predictors of *Ixodes scapularis* distribution across the models as measured by percent variance explained by each predictor variable by percent variance explained.

**FIGURE 2:**
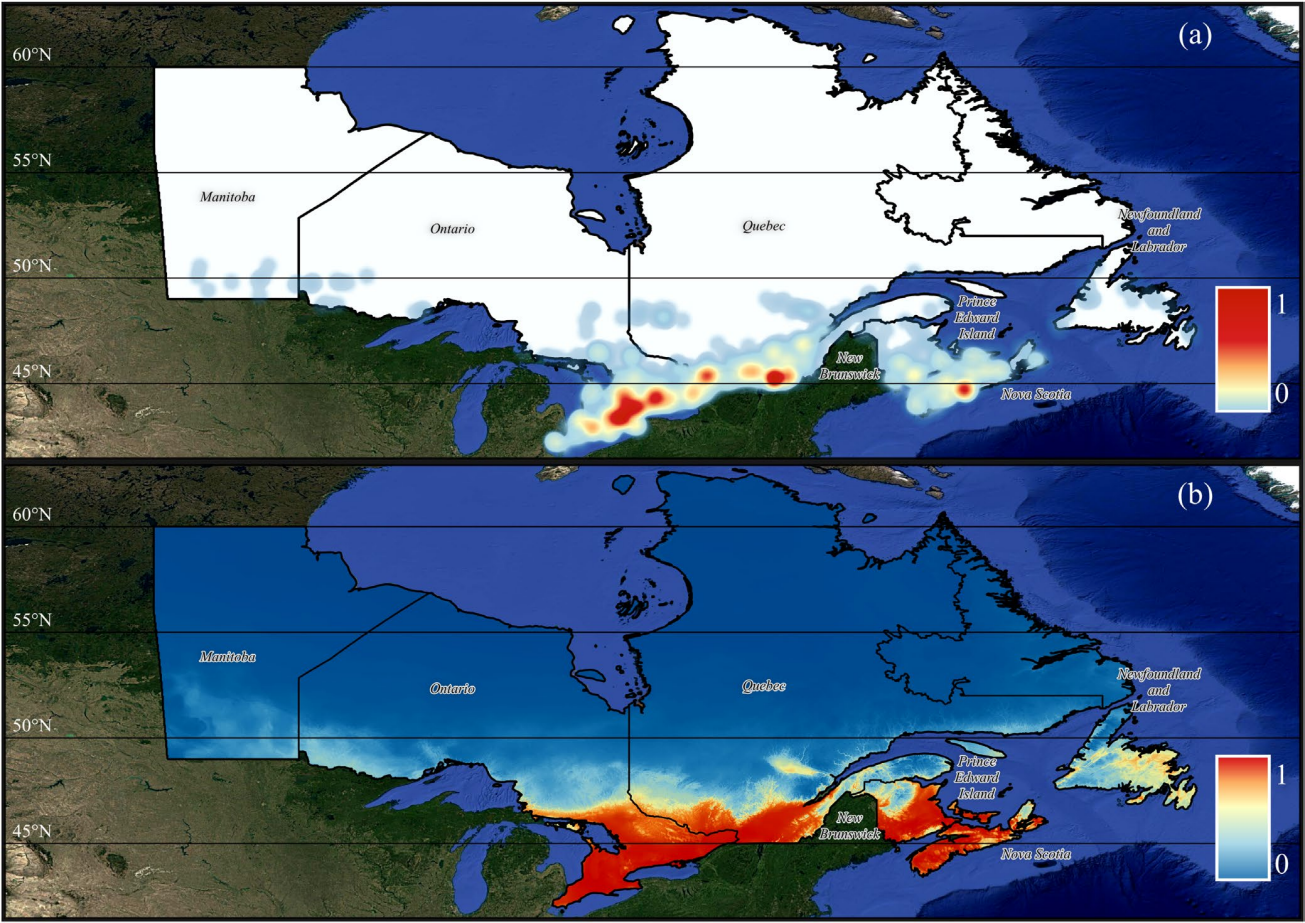
Heatmaps of the (a) empirical *I. scapularis* community occurrence sampling records shown by a relative density between 0 and 1 and (b) the historical *I. scapularis* ecological niche map created using observed environmental data from 1981 to 2010. In panel (b), niche suitability (*p*) is categorized as follows: Dark blue indicates unsuitable (*p* = 0–0.1), light blue represents low suitability (*p* = 0.1–0.3), yellow signifies medium suitability (*p* = 0.3–0.6), orange denotes high suitability (*p* = 0.6–0.8), and red marks optimal suitability (*p* = 0.8–1.0). Map lines delineate study areas and do not necessarily depict accepted national boundaries.

The ensemble model using the AdaptWest dataset identified several significant variables, including Degree Days Above 5°C (DD5), Hogg's Climate Moisture Index (CMI), Mean Annual Temperature (MAT), Mean Annual Precipitation (MAP), Winter Precipitation (PPT_wt), Degree Days Above 18°C (DD18), Degree Days Between 10°C and 40°C (DD1040), Precipitation as Snow (PAS), Degree Days Below 0°C (DD_0), and the Mean Extreme Maximum Temperature over the Past 30 Years (EXT; Figure S2). These findings, which highlight key variables in temperature and precipitation metrics, align closely with patterns observed in the ChelsaBioclim+ dataset. The resulting ensemble models exhibited similar patterns of range expansion, directional shifts, and hotspot locations but provided more conservative suitability and niche growth estimates. Net primary productivity (NPP, or biomass production), temperature, precipitation (especially during the driest periods), and the length of the growing season represent the primary variables influencing *I. scapularis* niche suitability (Figure 1). Using the empirical presence data (Figure 2a), our historical niche model for *I. scapularis* has identified regions that align closely with the species' recorded distribution used in our analysis (Figure 2b). An area totaling 321,003 km^2^ demonstrates optimal environmental conditions for *I. scapularis* , primarily concentrated in southern Ontario, Quebec, Nova Scotia, and New Brunswick regions. Similarly, under the SSP3‐7.0 scenario (Figure S3), high‐suitability areas remain largely consistent with SSP5‐8.5 projections. However, the rate and extent of change are more extreme under SSP5‐8.5, reflecting the stronger climate forcing. The transition from unsuitable to suitable conditions is more gradual, illustrated by a slower reduction in low‐suitability regions and a steadier emergence of moderate to high‐suitability zones.

**FIGURE 3:**
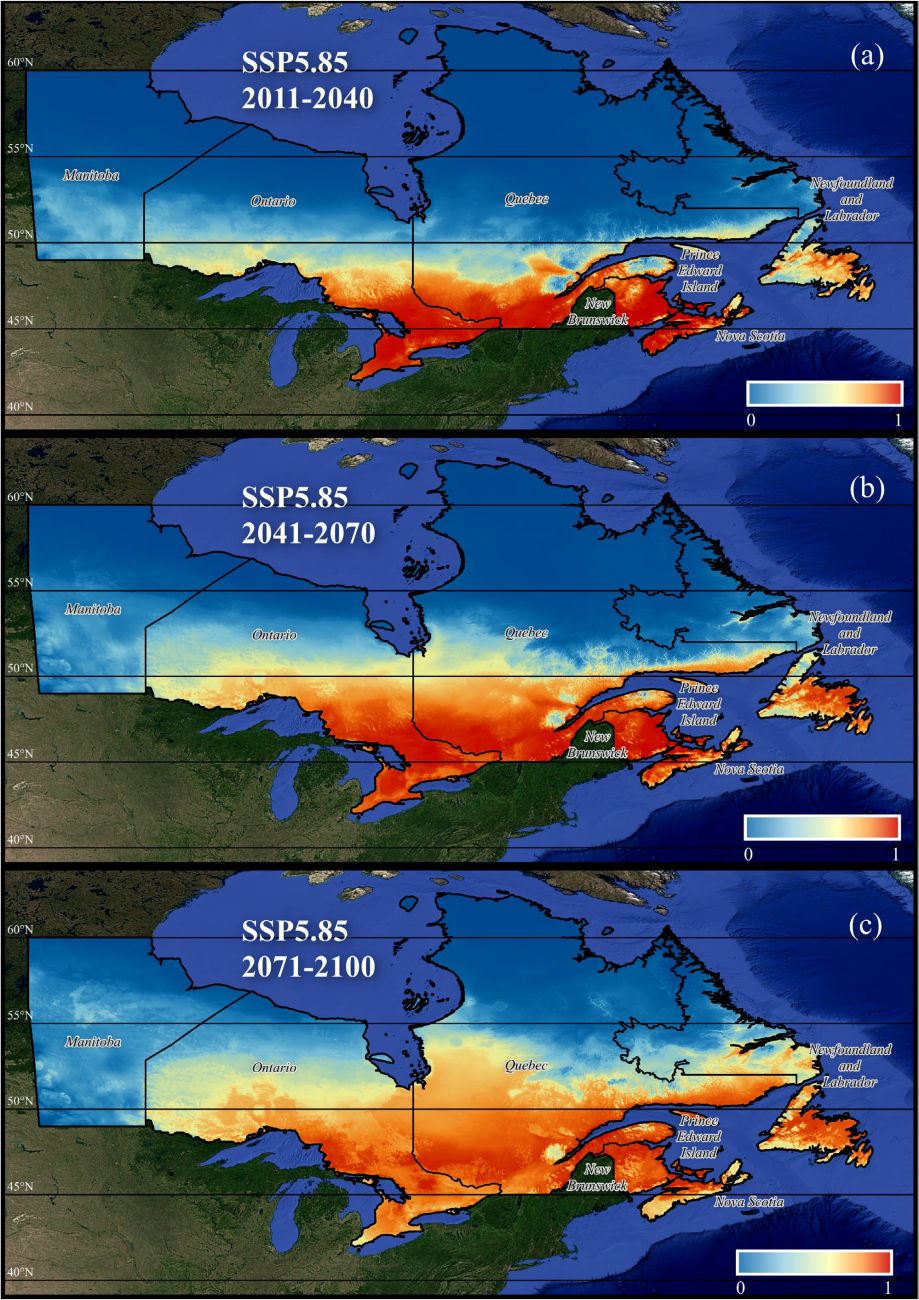
Projected changes in the ecological niche of *I. scapularis* under future climate conditions. The maps illustrate niche shifts based on SSP5‐8.5 climate simulation data for the periods (a) 2011–2040, (b) 2041–2070, and (c) 2071–2100. Niche suitability (*p*) is categorized as follows: Dark blue indicates unsuitable (*p* = 0–0.1), light blue represents low suitability (*p* = 0.1–0.3), yellow signifies medium suitability (*p* = 0.3–0.6), orange denotes high suitability (*p* = 0.6–0.8), and red marks optimal suitability (*p* = 0.8–1.0). Map lines delineate study areas and do not necessarily depict accepted national boundaries.

The data presented in Figure S4 shows how habitat suitability for *I. scapularis* changes in response to variations in key environmental variables (as shown in Figure 1). Suitability is highest in areas with net primary productivity (NPP) around 1250 gC m^−2^ year^−1^ (Figure S4a) and in regions where the annual thermal range falls between 5°C and 15°C, with habitat suitability dropping to near zero at mean annual temperatures below −10°C and above 25°C (Figure S4b). A growing season length (GSL) of approximately 250 days or more supports the highest suitability (Figure S4c). Temperature variables during the warmer season are further influential, with the mean temperature of the warmest quarter (Bio10) showing an optimal range between 20°C and 23°C (Figure S4e), and a similar but slightly less sensitive response observed for temperature variations within the warmest month (Bio5, Figure S4j). Regions with moderate to low intra‐annual temperature variation are preferable (Figure S4h). The “last growing degree day (GDD) above 10°C” variable (Figure S4i; measured in Julian days) showed relatively low sensitivity to Julian day with a broad spread between day ~100 and ~250. In terms of precipitation, *I. scapularis* is more responsive to seasonal moisture limitations than overall annual rainfall. Mean monthly precipitation amounts between 200 and 350 mm during the driest quarter (Bio17) are ideal (Figure S4d), while precipitation during the driest month (Bio14) is optimal within a range of 50 mm to 150 mm (Figure S4f). Annual precipitation (Bio12) is most favorable within the 1250 mm to 2500 mm range (Figure S4g). Ongoing alterations in these environmental drivers in particular will expand the tick's range sixfold, pushing its northern boundary approximately 500 km further north by the end of the century (Figure 3).

As environmental drivers such as temperature, precipitation, biomass production (NPP), and length of the growing season continue to change, we can expect notable range expansion of *I. scapularis* . By 2040, the total area of optimal habitat conditions will increase to 480,101 km^2^ (Figure 3a). By 2070, *I. scapularis* habitat could extend up to 664,554 km^2^ across eastern Canada, representing a nearly twofold increase in area compared to historic ranges, and reach as far as 51° N (Figure 3b). By 2100, the range of *I. scapularis* is projected to expand by an additional 4.23% (Figure 3c). This expansion is concentrated near the northern boundary of their 2070 range, indicating a continued northward shift. Conversely, a slight reduction in their southern distribution is anticipated related to increasing mean temperatures and more xeric conditions but could reflect the inherent uncertainties in modeling future projections.

**FIGURE 4:**
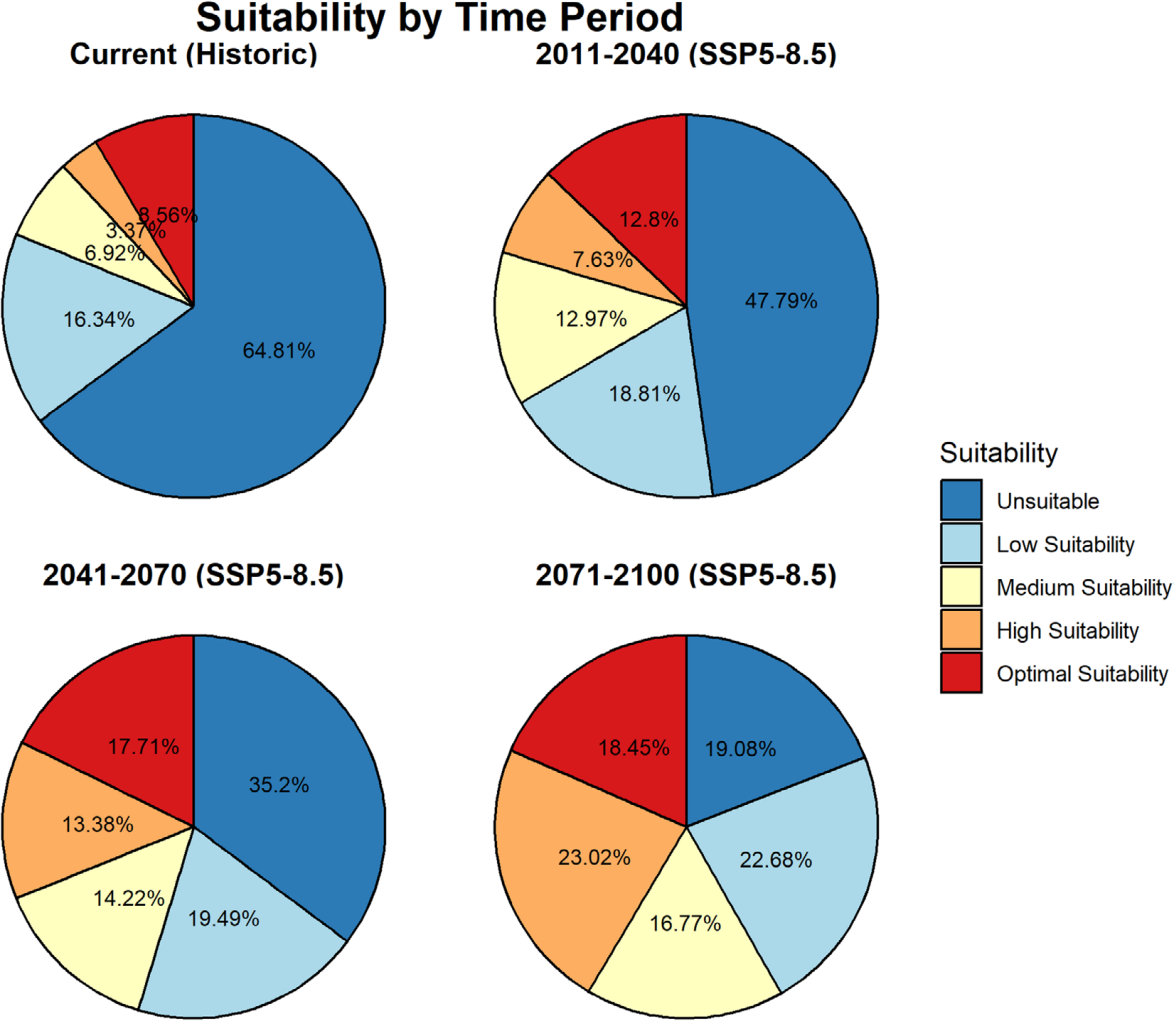
Pie charts showing the temporal changes in relative land suitability of *Ixodes scapularis* for current/historical and forecasted time periods under future climate scenarios (SSP5‐8.5) in eastern Canada.

Along with the distributional changes of *I scapularis*, our modeling also suggests that we could see significant changes in the proportions of suitable land area over time (Figure 4, *X* ^2^ = 54.877, *p* = 1.904e‐07). We particularly see a significant decrease in unsuitable area from the historical to 2100 (*X* ^2^ = 47.7, *p* = 4.967e‐12) and significant increases in medium, high and optimal suitability, respectively (*X* ^2^ = 6.6, *p* = 0.01; *X* ^2^ = 26.36, *p* = 2.833e‐07; *X* ^2^ = 5.85 *p* = 0.02) between these two time points. The projections under SSP3‐7.0 are very similar, particularly the changes in projected optimally suitable land area over the projected time points (Figure S5).

## 4 Discussion

We employed a suite of modeling approaches to create ensembled models to predict the present distribution and forecast the future distribution of *I. scapularis* in eastern and part of central Canada under climate change. While species distribution models have long been used as tools to inform conservation planning and policy, we apply these techniques to emphasize the escalating risk of *I. scapularis* exposure to communities at higher latitudes. Specifically, we aimed to highlight the latent threat of zoonotic diseases, whose prevalence is influenced by the distribution of infected vertebrate hosts.

Terrestrial arthropods, including ticks, demonstrate significant phenotypic plasticity in response to rising temperatures and changing moisture regimes, a phenomenon particularly evident in pest species. These changes frequently impact species' physiology, behavior, phenology, distribution, population dynamics, community composition, and interspecies interactions (Harvey et al. 2022). As a result, predicting species trajectories and effectively monitoring their responses become increasingly complex, but the use of several modeling approaches to create ensemble models is flexible and can help improve accuracy, robustness, and stability in prediction. As the distribution of *I. scapularis* expands northward, these pathogens, carried by both the ticks and their hosts pose a growing risk to public health. The current model predictions align with the known occurrence points. Future projections indicate that suitable habitats will expand to higher latitudes across most of eastern Canada, with existing climatic limitations (e.g., temperature and precipitation) in northern regions being surpassed across various climate scenarios over the coming decades.

In the 1970s, only one population of *I. scapularis* was known in southern Ontario, Canada (Watson and Anderson 1976). Today, numerous *I. scapularis* populations are present across Ontario, Quebec, Manitoba, Nova Scotia, Prince Edward Island and New Brunswick. Only recently have surveillance efforts yielded detection of *I. scapularis* populations in Saskatchewan and as far west as Alberta (Chilton et al. 2020; eTick 2022). Our research has identified several critical factors influencing habitat suitability for *I. scapularis* , including temperature, precipitation, biomass production, growing season length, the climate moisture index, and the annual accumulation of degree days. Our models predict that changes in these variables, among others, will cause *I. scapularis'* distribution to expand north from its current foci in southern Canada, facilitated by a high abundance of suitable hosts including mammal and bird reservoir species that harbor zoonotic, tick‐borne pathogens (Ogden, Trudel, et al. 2006). The northern front will tend eastward rather than toward the Canadian prairies, likely due to the latter's excessive dryness and aridity, conditions that are less optimal for *I. scapularis* and predicted to persist with climate change even if temperatures become more favorable for *I. scapularis* (Ogden, Trudel, et al. 2006). The mass migration of birds each spring into Canada aids this expansion, passing through the north‐central and northeastern United States where there is a high prevalence of *I. scapularis* (Ogden, Trudel, et al. 2006). The long‐range dispersal of birds, and the diseases for which they serve as reservoirs, facilitates the expansion of tick ranges without the constraints of ecological connectivity and land cover (Scott et al. 2018; Tardy et al. 2023) and may help explain the probability of tick introduction. The southern trailing edge of the range will consolidate in southern Ontario, Quebec, New Brunswick and Nova Scotia.

In southeastern Manitoba, *I. scapularis* populations are established (Krakowetz et al. 2011); however, our baseline models show low suitability for that region. Projections for future climate scenarios suggest that Manitoba will remain a low‐suitability area, though we predict some northern expansion from unsuitable to low‐suitability regions. The persistence of ticks in this region, despite the models' predictions, could be attributed to localized influences, such as the complex nature of microclimates, which play a crucial role in tick survival but are often not fully captured in broader‐scale climatic assessments (Ogden, St‐Onge, et al. 2008).

Similarly, Leighton et al. (2012) employed an empirical model of the rapid range expansion by *I. scapularis* into Canada. They highlighted that temperature (growing degree days) was the most important predictor of the northward shift in the distribution of the tick using empirical passive surveillance data. Our projections also map very closely to that of the Institute of Public Health in the province of Québec (2024), which also uses eTick data. This is particularly true under the SSP3‐7.0 scenario; however, our modeling shows a much broader distribution across the province and eastern Canada, especially at higher latitudes. However, our projections do indicate an expansion of suitable habitat for *I. scapularis* along the northern St. Lawrence River—findings consistent with those of Ripoche et al. (2022), who similarly projected increasing tick populations in the region. These projections are supported by recent active surveillance data confirming the presence of *I. scapularis* north of the St. Lawrence River, where they had previously been detected only south of the river (Ripoche et al. 2022).

Temperature and precipitation (relating to humidity) play a crucial role in the prevalence and survival of *I. scapularis* (Schulze et al. 2009; Nielebeck et al. 2023). Our research shows precipitation during dry periods and the growing season is significant. Within each morphological phase, *I. scapularis* must balance wet and dry conditions, with winter and spring being particularly critical. Mild, wet winters boost survival rates (Hayes et al. 2015; Nielebeck et al. 2023) and increased spring precipitation supports questing and longevity (McCabe and Bunnell 2004). Snow and leaf litter accretion insulates ticks from the cold, further enhancing survival (Templer et al. 2012; Burtis and Pflueger 2017; Linske et al. 2019). However, climate change‐induced reductions or delays in winter snowpack could increase mortality (Templer et al. 2012). Additionally, increased rainfall stabilizes temperatures and raises moisture content in forest duff, improving tick habitats (McCabe and Bunnell 2004), while extreme rainfall may hinder survival and expansion (Ogden, Maarouf, et al. 2006). Warmer, drier climates cause rapid water loss in *I. scapularis* (Nielebeck et al. 2023). This could have major implications for the southern distribution of ticks where we might expect range contractions in conjunction with ongoing northern range expansion in this species. Future modeling efforts might consider incorporating tick host and *B. burgdorferi* presence in more northern areas.

Temperature is a critical determinant of *I. scapularis* distribution (Clark 1995; Brunner et al. 2012; Tardy et al. 2023). These ticks cannot survive prolonged exposure to temperatures at −10°C, with lethal thresholds ranging from −11.0°C to −16.0°C (Lindsay et al. 1998; Brunner et al. 2012) or above 30°C (Eisen and Eisen 2024). In our models, the optimal annual mean temperature range for *I. scapularis* is approximately 6.0°C to 12.0°C (±1.0°C), aligning with activity thresholds of 6.2°C ± 3.6°C for nymph females and 8.5°C ± 3.0°C for nymph males (Clark 1995). Additionally, uncoordinated activity thresholds, the temperature below which a tick can no longer seek a host in a coordinated manner, are 9.2°C ± 4.1°C for nymphal females and 11.2°C ± 3.4°C for nymphal males (Clark 1995). However, it is worth noting that laboratory studies on the cold hardiness of *I*. *scapularis* indicate that the species requires temperatures above 16°C with high humidity for at least part of the year to complete its life cycle (Eisen and Eisen 2024). Female oviposition is inhibited below 6°C–8°C, and egg masses fail to produce larvae below 12°C. Molting of fed larvae is inhibited below 12°C and below 16°C in nymphs (Eisen and Eisen 2024). Higher temperatures (within a range of 8°C–28°C for oviposition and 12°C–28°C for larval hatching) accelerate these processes (Eisen and Eisen 2024).

The duration during which temperatures remain above critical thresholds is a key determinant of habitat suitability. Our ensembles suggest that *I. scapularis* find regions with a higher amount of below‐freezing degree days unfavorable, while warmer climates are associated with an accelerated life cycle and increased reproductive rates within tick populations (Eisen et al. 2016). The models suggest an extended growing season significantly enhances habitat suitability, notably if it exceeds 200 days. *I. scapularis* can complete its life cycle under optimal conditions in fewer than 230 days (Eisen et al. 2016). Eastern Canada's growing season typically spans 80 to 140 days (Natural Resources Canada 2020). Climate change contributes to earlier spring start dates and later fall end dates, with projections suggesting an extension of the growing season by 20 to 40 days uniformly across Canada throughout the 21st century (Natural Resources Canada 2020). These changes are anticipated to accelerate developmental rates and increase activity and could change seasonal synchrony in *I. scapularis* , which could have implications for co‐feeding transmission between life stages, increasing tick‐borne pathogen circulation in the local community. Our models suggest that the relationship between growing degree days (GDD) and *I. scapularis* is significant because the GDD and the growing season occur during spring and summer. This period coincides with resumed activity (i.e., from March to April) and development (i.e., from May to September) in *I. scapularis* (Lindsay et al. 1998).

Net primary production (NPP) is a measure of annual plant biomass production and can serve as an indicator of ecosystem productivity. Our models suggest that *I. scapularis* is most likely to occupy regions with intermediate biomass production, such as temperate forests and grasslands and exhibits a preference for areas with higher biomass concentrations. While we could not explicitly capture land cover metrics in this analysis, the boreal biome has not been historically suitable tick habitat; however, the forest is also changing rapidly and several studies have shown a shift towards more broadleaf/temperate forest, particularly at the low‐mid‐ boreal latitudes (Boisvert‐Marsh et al. 2014; Brice et al. 2019; Boisvert‐Marsh and de Blois 2021). Canada's forested regions encompass extensive temperate and boreal zones. Predicted increases in NPP in temperate and boreal forests in response to climate change (Melillo et al. 1993) could further increase their suitability.

We analyzed two high‐priority emission scenarios to explore how varying climate trajectories might influence the spread of *I. scapularis* . We focused on the high‐emission SSP5‐8.5 scenario because it is more prudent from a risk management perspective to overestimate the potential range of *I. scapularis* rather than underestimate the risks of increased exposure (Soucy et al. 2018). Under the SSP3‐7.0 scenario, the projected range expansion of *I. scapularis* from 2011 to 2040 parallels that of the SSP5‐8.5 scenario, with the resulting distribution maps displaying virtually indistinguishable changes. Minor variations were observed in the extent of optimal suitable areas and hotspots, measuring 480,101 km^2^ compared to 457,481 km^2^. From 2040, no new notable focal areas emerge; instead, expansions occur from existing foci and reach higher latitudes of 50° N by 2070. This pattern is consistent across both the SSP5‐8.5 and SSP3‐7.0 scenarios (599,536 km^2^ vs. 664,554 km^2^, respectively). By 2100, we project the range of *I. scapularis* to extend to 52° N under the SSP3‐7.0 scenario and to 53° N under the more trying SSP5‐8.5 scenario. However, despite the broader latitudinal expansion in SSP5‐8.5, the optimal suitable habitat is projected to be larger under SSP3‐7.0, encompassing 712,587 km^2^ to 692,671 km^2^ respectively in the higher‐emission scenario. Future modeling efforts might incorporate biotic predictors (e.g., host/reservoir abundance) to improve model predictions.

Intervention strategies are employed to mitigate the transmission of tick‐borne diseases. These methods typically aim to lower tick density in the environment using acaricides, reducing the prevalence of infections in hosts and ticks, or minimizing the interactions between humans and ticks (Ruiz‐Carrascal et al. 2024). Canadian health information websites from government and patient groups often address personal protection, transmission, and tick identification in Lyme disease prevention; however, inconsistencies and misinformation sometimes emerge in online content (Journault et al. 2020). Compounding this issue is the ongoing challenge of developing cost‐effective, low‐effort, and environmentally sustainable interventions that ensure safety for both humans and animals (Eisen 2021). Our findings on the expanding range of *I. scapularis* highlight the elevated threat of novel species interactions and increased human‐tick exposures in more northern regions. In the years following our empirical data used for modeling, more *I. scapularis* records have been reported in the western part of Canada and further north than previously (eTick 2025). These results underscore the importance of implementing proactive tick control and public health communication (especially to northern communities) and interventions during a critical period in which the range of *I. scapularis* continues to move northward. This is relevant not only to the control and prevention of Lyme disease, but also to a suite of other diseases (e.g., anaplasmosis, babesiosis) vectored by this tick. Our findings, when integrated with traditional tick monitoring methods (Tiffin et al. 2022), can be used to help authorities (e.g., public health) with crucial contouring of likely at‐risk areas, enabling the efficient allocation of resources and targeted awareness campaigns in at‐risk areas.

## Author Contributions

**Jacob R. Westcott:** conceptualization, data curation, formal analysis, investigation, methodology, validation, visualization, writing – original draft, writing – review and editing. **Joseph J. Bowden:** conceptualization, investigation, methodology, project administration, resources, supervision, validation, writing – original draft, writing – review and editing. **Jade Savage:** investigation, validation, writing – original draft, writing – review and editing. **Karen M. Doody:** conceptualization, investigation, project administration, supervision, writing – original draft, writing – review and editing.

## Conflicts of Interest

The authors declare no conflicts of interest.

## Supporting information


**Data S1:** gcb70591‐sup‐0001‐DataS1.pdf.

## Data Availability

The data and code that support the findings of this study are openly available in Zenodo at http://doi.org/10.5281/zenodo.14675093 and http://doi.org/10.5281/zenodo.14682898, respectively. ERA5 observational data were obtained from the Copernicus Climate Change Service (C3S) Climate Data Store (CDS) at https://doi.org/10.24381/cds.adbb2d47. CMIP6 outputs were obtained from the Inter‐Sectoral Impact Model Intercomparison Project (ISIMIP) database via Zenodo at https://doi.org/10.5281/zenodo.4686991. The E3SM model (ELMv2) can be accessed from DOE CODE at https://doi.org/10.11578/E3SM/dc.20210927.1 and Github at https://github.com/E3SM‐Project/E3SM/releases/tag/v2.0.0.
