# Supplementary material for: Rapid Northward Expansion of the Blacklegged Tick, Ixodes scapularis , in Response to Climate Change
Source: Glob Chang Biol. 2025 Nov 6;31(11):e70591. doi: 10.1111/gcb.70591 (PMC12590538; doi:10.1111/gcb.70591)
Supplement: Supplementary file 1 — Data S1: gcb70591‐sup‐0001‐DataS1.pdf. [file GCB-31-e70591-s001.pdf]

SUPPLEMENTARY INFORMATION

Supplementary Figures

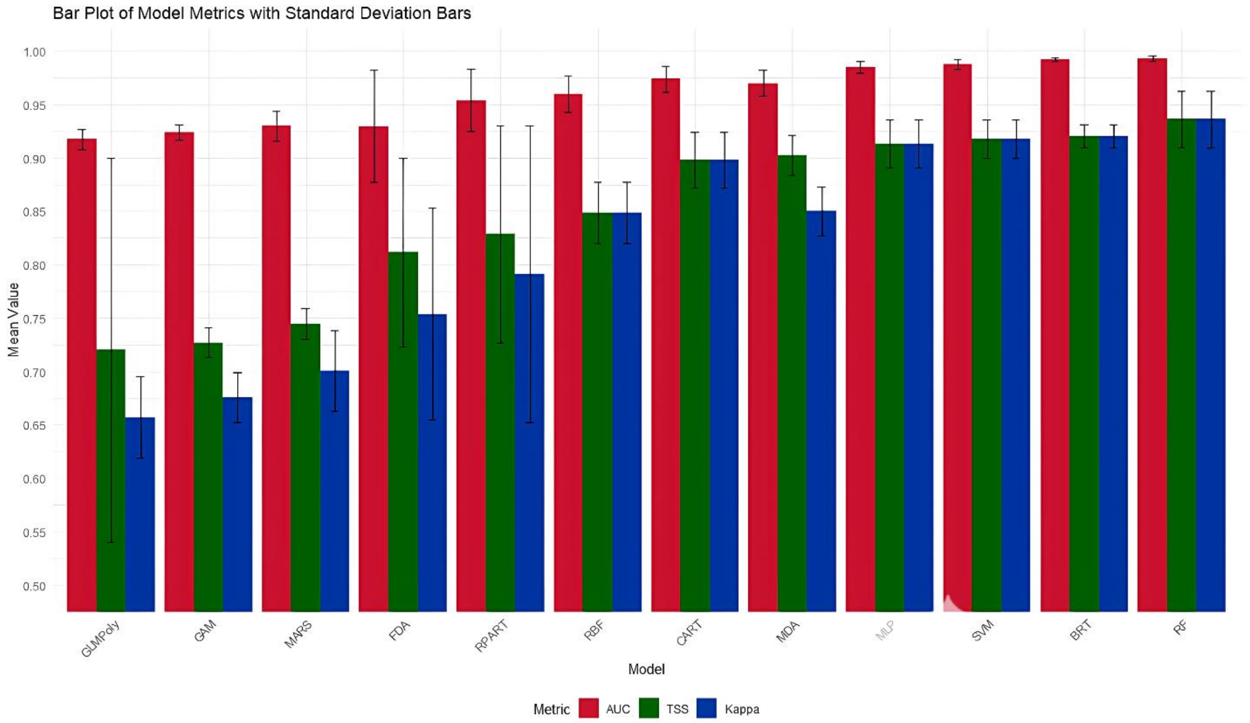

Figure S1: Summary statistics and standard deviations between the 12 algorithms used to assess model performance. AUC (Red), TSS (Green) and Kappa (Blue) are performance metrics to evaluate how well each algorithm used predicts the presence or absence of I. scapularis. AUC and TSS are used to evaluate the ability of models to distinguish between presence and absence. TSS is particularly well-suited for binary classification tasks and imbalanced data. Kappa accounts for agreement expected by chance and is effective at dealing with uncertainty or variations in prevalence.

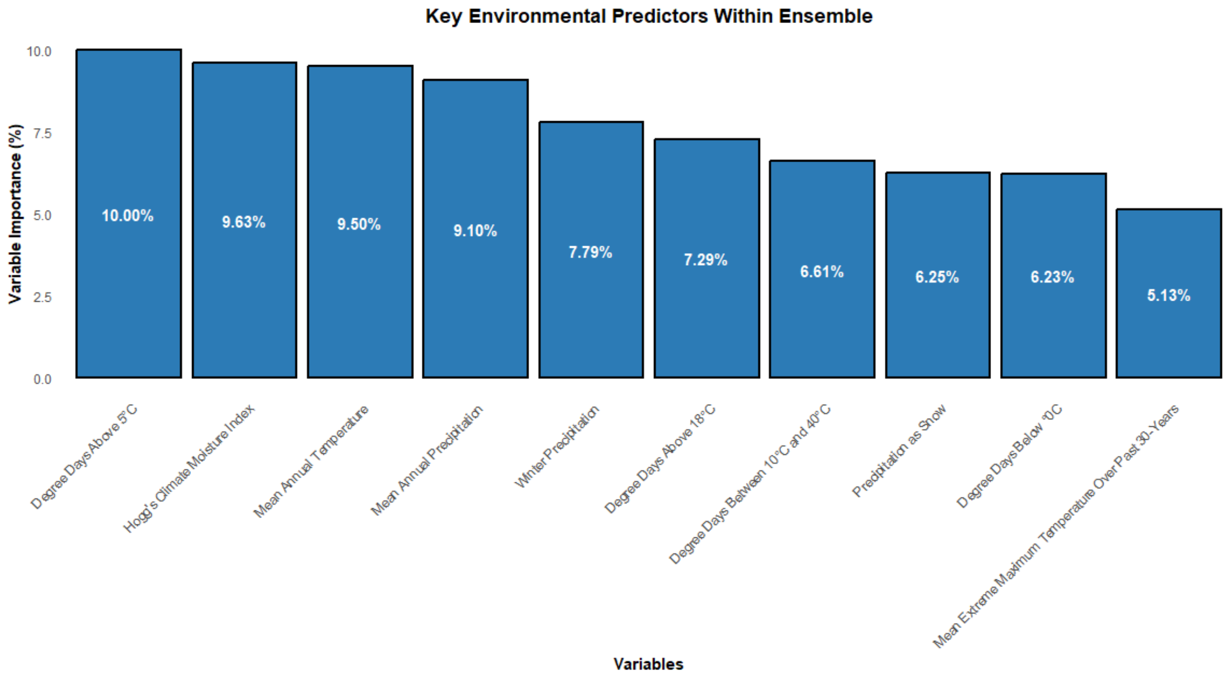

Figure S2: The most influential AdaptWest environmental predictors of Ixodes scapularis distribution across the models as measured by percent variance explained by each predictor variable by percent variance explained.

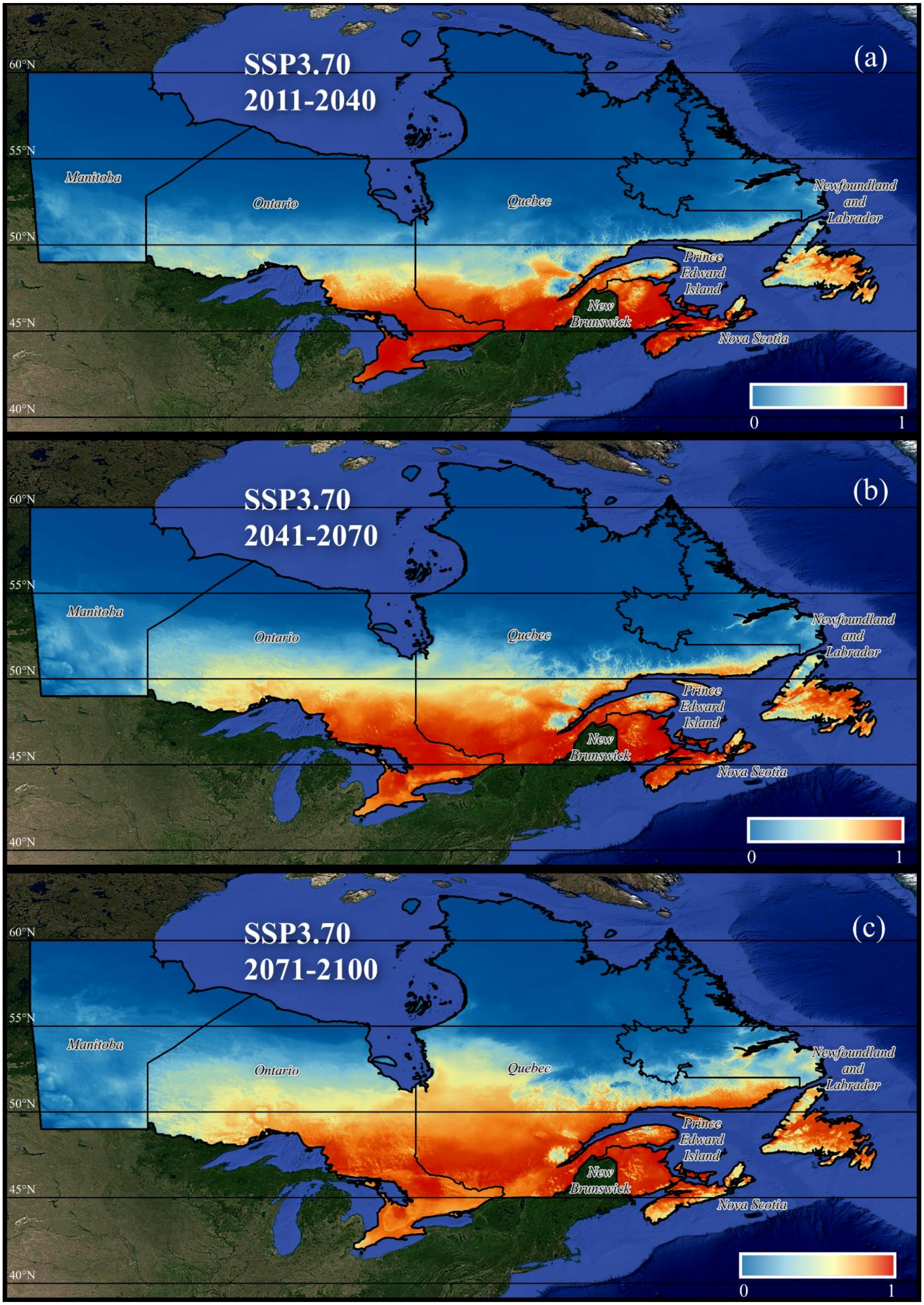
SSP3.70
2011-2040
(a)
60°N
55°N
50°N
45°N
40°N
Manitoba
Ontario
Quebec
Newfoundland and Labrador
Prince Edward Island
New Brunswick
Nova Scotia
0
1
SSP3.70
2041-2070
(b)
60°N
55°N
50°N
45°N
40°N
Manitoba
Ontario
Quebec
Newfoundland and Labrador
Prince Edward Island
New Brunswick
Nova Scotia
0
1
SSP3.70
2071-2100
(c)
60°N
55°N
50°N
45°N
40°N
Manitoba
Ontario
Quebec
Newfoundland and Labrador
Prince Edward Island
New Brunswick
Nova Scotia
0
1

Figure S3: Projected changes in the ecological niche of I. scapularis under SSP3.70 climate scenarios for the periods (a) 2011-2040, (b) 2041-2070, and (c) 2071-2100. Niche suitability (p) is categorized as follows: dark blue indicates unsuitable (p = 0 - 0.1), light blue represents low suitability (p = 0.1 - 0.3), yellow signifies medium suitability (p = 0.3 - 0.6), orange denotes high suitability (p = 0.6 - 0.8), and red marks optimal suitability (p = 0.8 - 1.0). Map lines delineate study areas and do not necessarily depict accepted national boundaries

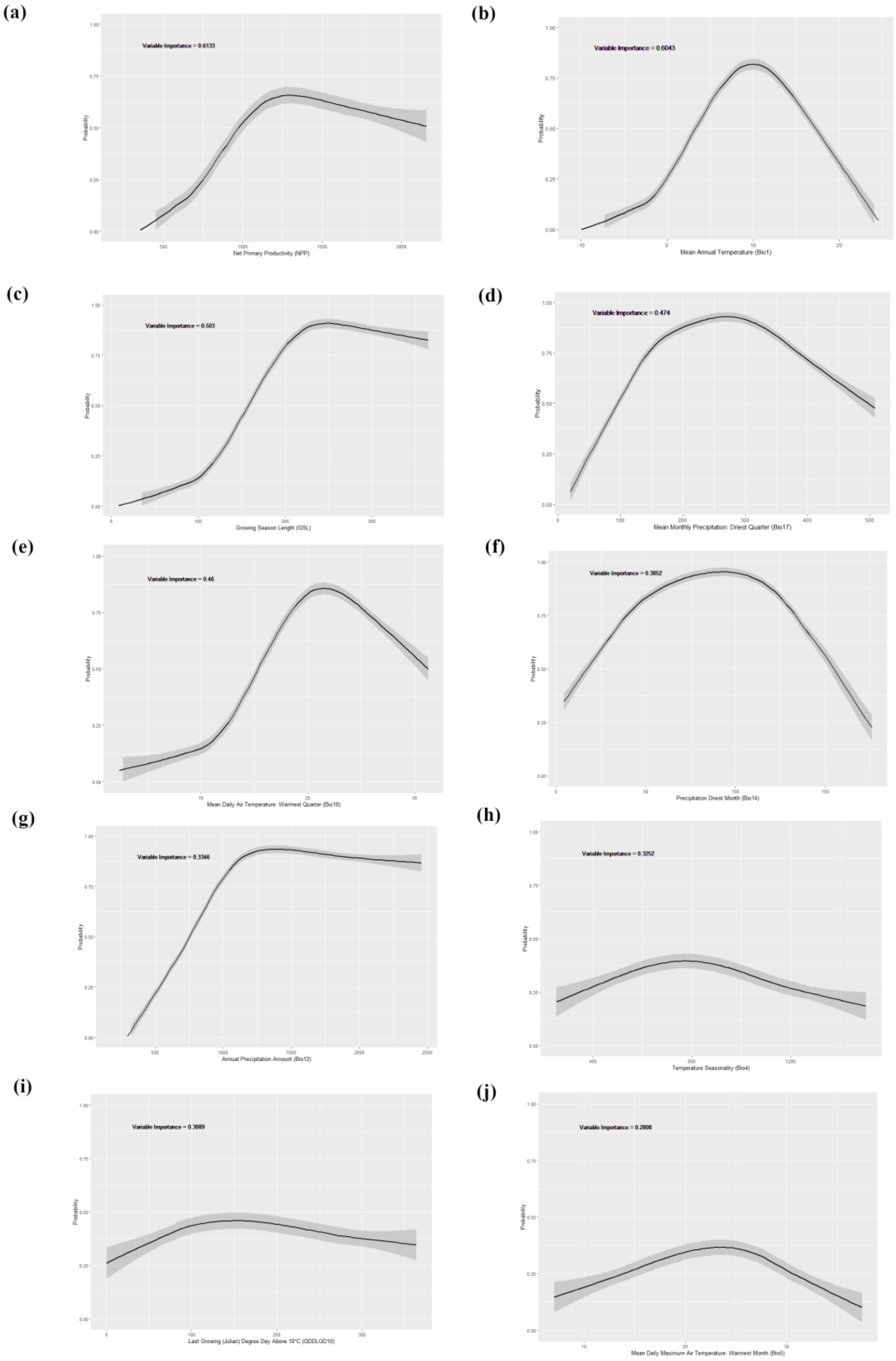
(a)
Variable Importance = 0.6133
Probability
Net Primary Productivity (NPP)
(b)
Variable Importance = 0.6043
Probability
Mean Annual Temperature (Bio1)
(c)
Variable Importance = 0.503
Probability
Growing Season Length (GSL)
(d)
Variable Importance = 0.474
Probability
Mean Monthly Precipitation: Driest Quarter (Bio17)
(e)
Variable Importance = 0.46
Probability
Mean Daily Air Temperature: Warmest Quarter (Bio10)
(f)
Variable Importance = 0.3852
Probability
Precipitation Driest Month (Bio14)
(g)
Variable Importance = 0.3346
Probability
Annual Precipitation Amount (Bio12)
(h)
Variable Importance = 0.3252
Probability
Temperature Seasonality (Bio4)
(i)
Variable Importance = 0.3089
Probability
Last Growing (Julian) Degree Day Above 10°C (GDDLGD10)
(j)
Variable Importance = 0.2808
Probability
Mean Daily Maximum Air Temperature: Warmest Month (Bio5)

Figure S4: Response curves for our models' ten most influential environmental variables associated with I. scapularis distribution. Each curve illustrates the modelled relationship between a single environmental predictor and the probable habitat suitability for I. scapularis while holding all other covariates constant.

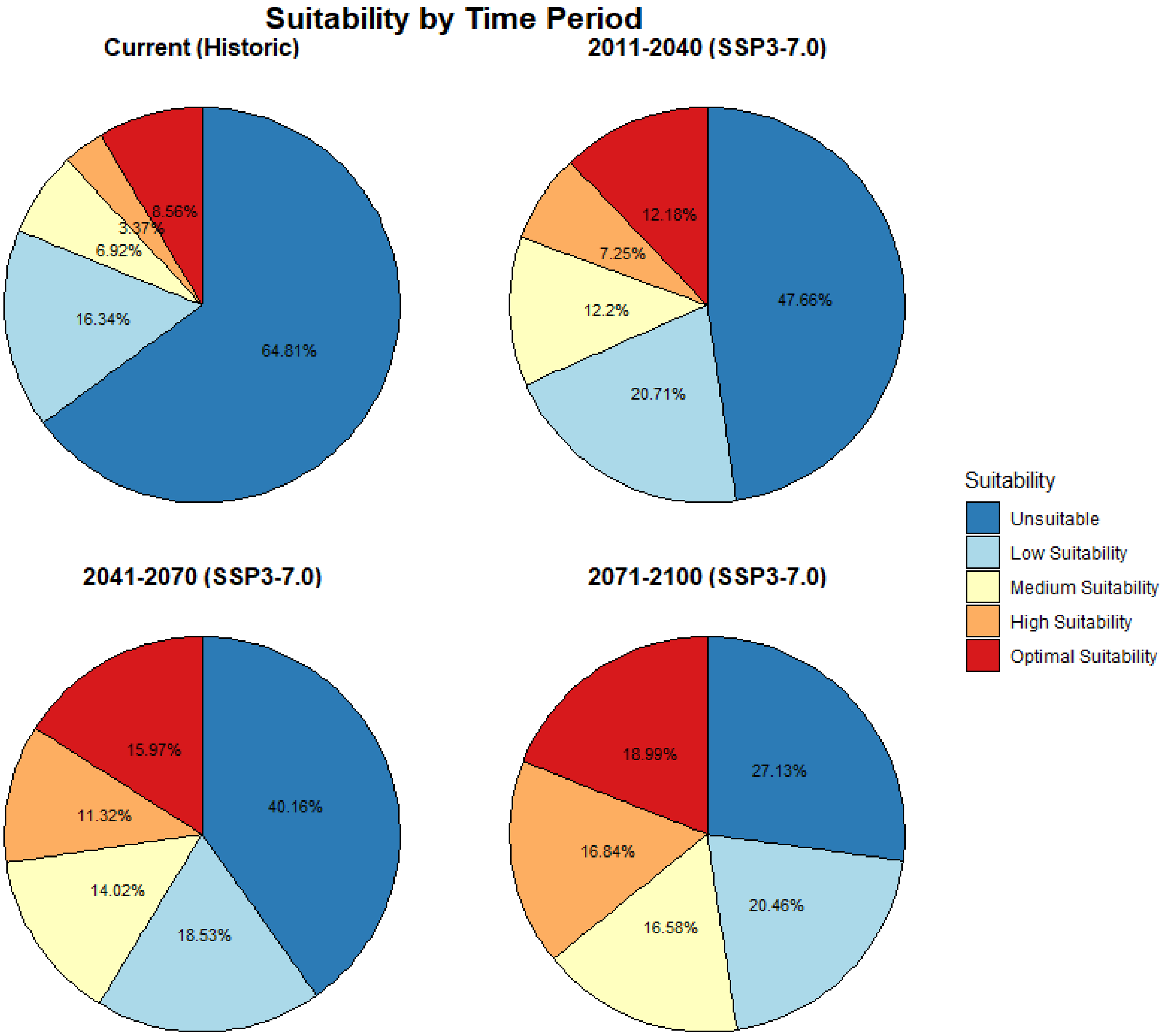

Figure S5: Pie charts showing the temporal changes in relative land suitability of Ixodes scapularis for current/historical and forecasted time periods under future climate scenarios (SSP3-7.0) in eastern Canada.

Supplementary Tables

Table S1: The list of States with suitable *Ixodes scapularis* records in the USA for our species distribution modelling analysis

#	State
1	Alabama
2	Arkansas
3	Connecticut
4	Delaware
5	Florida
6	Georgia
7	Illinois
8	Indiana
9	Iowa
10	Kansas
11	Kentucky
12	Louisiana
13	Maine
14	Maryland
15	Massachusetts
16	Michigan
17	Minnesota
18	Mississippi
19	Missouri

20	Nebraska
21	New Hampshire
22	New Jersey
23	New York
24	North Carolina
26	Ohio
27	Oklahoma
28	Pennsylvania
29	Rhode Island
30	South Carolina
31	South Dakota
32	Tennessee
33	Texas
34	Vermont
35	Virginia
36	West Virginia
37	Wisconsin

Table S2: AdaptWest Variables used in modelling:

Degree Days Above 5°C
Hogg's Climate Moisture Index
Mean Annual Temperature
Mean Annual Precipitation
Winter Precipitation
Degree Days Above 18°C
Degree Days Between 10°C and 40°C
Precipitation as Snow
Degree Days Below °0C
Mean Extreme Maximum Temperature Over Past 30-Years
Annual Heat Moisture
Spring Precipitation
Autumn Precipitation
Summer Heat Moisture
Temperature Difference between Warmest and Coldest Month (i.e., Measure of Continentality)
Summer Precipitation
Hargreaves Climate Moisture Index
Annual Relative Humidity
Elevation

Chelsa-Bioclim+ Variables used in modelling:

Net Primary Productivity
Mean Annual Temperature

Growing Season Length
Mean Monthly Precipitation - Driest Quarter
Mean Daily Air Temperature - Warmest Quarter
Precipitation - Driest Month
Annual Precipitation
Temperature Seasonality
Last (Growing Degree) Day above 10°C
Maximum Daily Mean Air Temperature - Warmest Month
Growing Season Precipitation
Last (Growing Degree) Day above 0°C
Last (Growing Degree) Day above 5°C
Precipitation Seasonality
Mean Diurnal Air Temperature Range
Isothermality
Snow-Water Equivalent
Frost-Cover-Frequency
Growing Season Temperatures
Annual Air Temperature Range
Mean Monthly Precipitation - Wettest Quarter
Mean Daily Air Temperature - Wettest Quarter
Precipitation - Wettest Month
